# Synopses of Editorial Board

**DOI:** 10.1080/13577149977668

**Published:** 1999-09


*Sarcoma* (1999) 3, 69–72

SYNOPSES OF EDITORIAL BOARD

### Nora Janjan, M.D., FACP

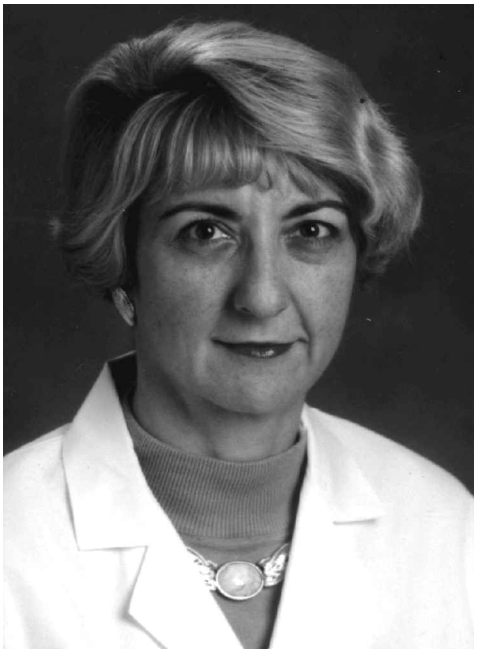

Radiation therapy, because it effectively treats microscopic residual disease, has allowed multi-modality approaches that expand the criteria for organ preservation. My research efforts in soft tissue sarcoma have focused on improving rates of local control and limiting normal tissue toxicities by optimizing radiotherapeutic approaches with brachytherapy and intraoperative irradiation. Brachytherapy has been used for definitive therapy of sarcomas that are newly diagnosed or that have recurred within a previously irradiated area. Despite concerns about wound healing and radiation effects on small vessels, we have shown that the viability of myocutaneous flap reconstruction is not compromised by brachytherapy. Providing comparable rates of local control, brachytherapy also proved to be an efficient, cost-effective alternative to conventional external beam radiation. Function preservation is an integral issue in quality of life, especially among patients with recurrent sarcoma in which limb-sparing surgical interventions are often facilitated by brachytherapy. Intraoperative irradiation and other techniques to exclude the small bowel from the external beam radiation portal have been applied to reduce risk of local recurrence in retroperitoneal sarcomas. Supportive care, that involves control of disease and treatment-related symptoms, has also been a research priority.

1357-714X print/1369-1643 online/99/020069-04 © 1999 Taylor & Francis Ltd

### Marc Horowitz, M.D.

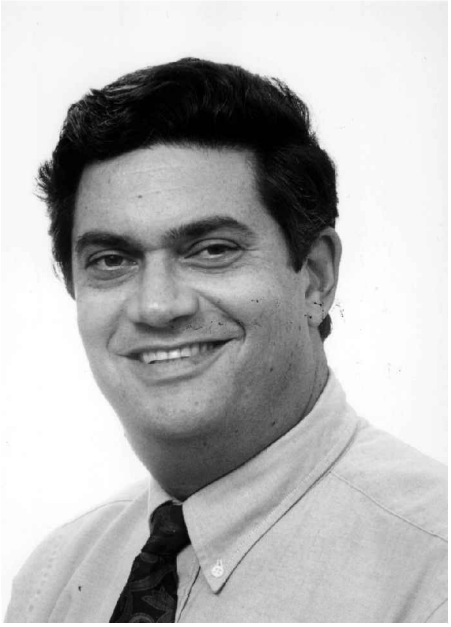

Marc Horowitz, M.D., is a Professer of Pediatrics at Baylor College of Medicine. He graduated from the University of Southern California School of Medicine and completed his postgraduate training at the Children's Hospital of Los Angeles, University of California at San Diego and the National Cancer Institute. He has published extensively in the area of development of new therapy for children with solid tumors including those of the central nervous system and bone and soft tissue sarcomas. He is Director of the Texas Children's Cancer Center's Solid Tumor Program and is responsible for all of the clinical and research activities relating to children with solid tumors. His clinical research activities include the development of protocols for children with solid tumors including studies of osteosarcoma, Ewing's sarcoma, neuroblastoma and high- and low-grade glioma, as well as gene therapy for recurrent brain tumors. Dr. Horowitz is also interested in medical informatics and is a developer of an electronic patient record that is in use at the Texas Children's Cancer Center.

### Professor Ann Barrett

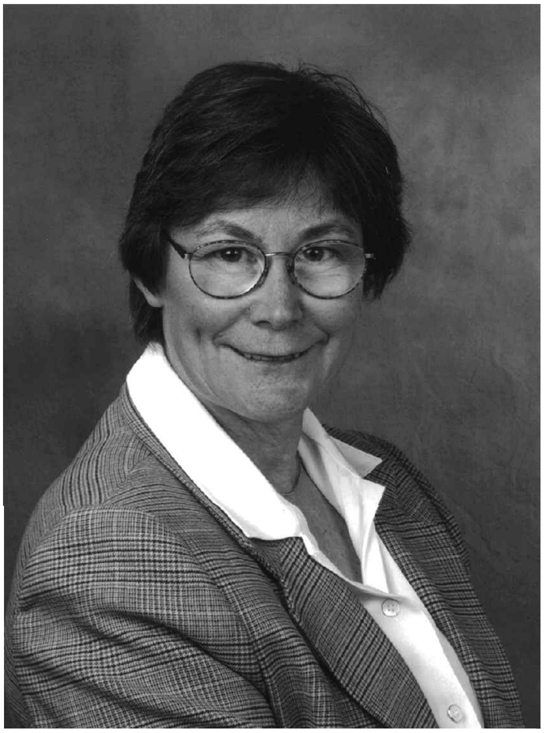

Professor Barrett is one of the Multi-disciplinary Bone and Soft Tissue Sarcoma Group at the Beatson Oncology Centre, Western Infirmary, Glasgow. She is the co-author of two books, *Practical Radiotherapy Planning* and *Cancer in Children* and is currently the President of the European Society of Therapeutic Radiation Oncology.

### Dr. A. N. (Bert) van Geel

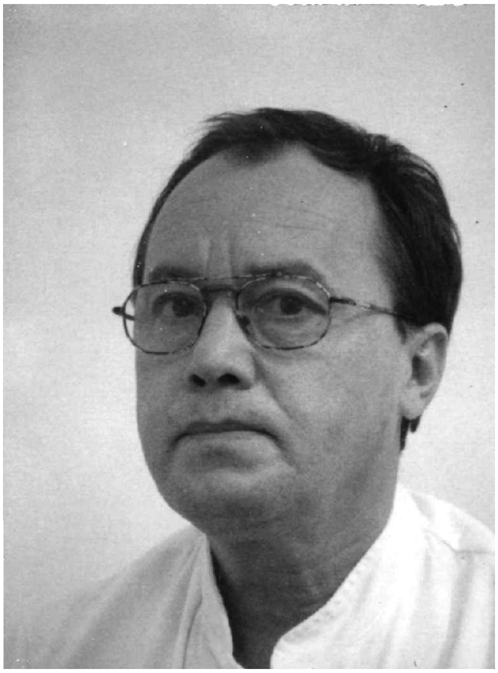

Bert van Geel was born in 1947 and became a surgeon in 1984. In that year he started his work as a surgical oncologist in the Daniel den Hoed Cancer Center (formally Rotterdam Radiotherapeutic Institute). The main goal was to found a surgical department essential for a good running cancer center. Nowadays he is mainly interested in thoracic and sarcoma surgery. One of the achievements is that he was the founder of the national sarcoma group (nederlandse werkgroep weke delen tumoren) and that this group was able to define a national concensus in the diagnosis and treatment of sarcoma. He is an active member of the local treatment group of the EORTC soft tissue and bone sarcoma group and the CTOS.

